# The role of cognitive load in modulating social looking: a mobile eye tracking study

**DOI:** 10.1186/s41235-020-00242-5

**Published:** 2020-09-16

**Authors:** Laura J. Bianchi, Alan Kingstone, Evan F. Risko

**Affiliations:** 1 University of Waterloo, Waterloo, ON N2L 3G1 Canada; 2 University of British Columbia, Vancouver, BC V6T 1Z4 Canada

**Keywords:** Social attention, Cognitive load, Eye-tracking, Gaze, Pedestrian passing

## Abstract

The effect of cognitive load on social attention was examined across three experiments in a live pedestrian passing scenario (Experiments 1 and 2) and with the same scenario presented as a video (Experiment 3). In all three experiments, the load was manipulated using an auditory 2-back task. While the participant was wearing a mobile eye-tracker, the participant’s fixation behavior toward a confederate was recorded and analyzed based on temporal proximity from the confederate (near or far) and the specific regions of the confederate being observed (i.e., head or body). In Experiment 1 we demonstrated an effect of cognitive load such that there was a lower proportion of fixations and time spent fixating toward the confederate in the load condition. A similar pattern of results was found in Experiment 2 when a within-subject design was used. In Experiment 3, which employed a less authentic social situation (i.e., video), a similar effect of cognitive load was observed. Collectively, these results suggest attentional resources play a central role in social attentional behaviors in both authentic (real-world) and less authentic (video recorded) situations.

## Significance statement

We found that cognitive load reduced looking toward a social agent, in both a live and video recorded pedestrian passing scenario. These results are consistent with the notion that looks toward social agents represent a potential attentional burden that may be avoided in cognitively demanding situations.

## Introduction

How individuals select locations and objects to attend to has long interested researchers in psychology (e.g., Itti & Koch, 2000; Triesman & Gelade, 1980; Wolfe, 1994). One area of research within this larger program that has attracted much interest recently is the examination of attention in social situations and in response to social stimuli (for reviews, see Birmingham & Kingstone, 2009; Emery, 2000). Understanding social attention provides important insights into our inherently social nature and the potential consequences of impairments in these processes (e.g., autism; Dawson et al., 2004; Ristic et al., 2005). In the present investigation we extend this work by examining the influence of cognitive load on social attention across more authentic (i.e., live) and less authentic (i.e., video recorded) social contexts using mobile eye tracking.

### The dual function of gaze framework

Social attention is often studied by examining when, where, and how an individual directs their attention toward or in response to social stimuli (e.g., people; Emery, 2000; Farroni, Massaccesi, Pividori, & Johnson, 2004; Gallup, Chong, Kacelnik, Krebs, & Couzin, 2014; Hayward, Voorhies, Morris, Capozzi, & Ristic, 2017; Kingstone, 2009; Laidlaw, Risko, & Kingstone, 2012; Latinus et al., 2015; Patterson, Webb, & Schwartz, 2002; Risko, Laidlaw, Freeth, Foulsham, & Kingstone, 2012; Risko, Richardson, & Kingstone, 2016; Tomasello, Hare, Lehmann, & Call, 2007). Interestingly, much social attention research is conducted in simple, putatively nonsocial contexts where individuals might be asked to look at schematic faces (Barton, Radcliffe, Cherkasova, Edelman, & Intriligator, 2006; Driver et al., 1999) or images of social scenes (Birmingham, Bischof, & Kingstone, 2008). As a result, this research has been criticized for failing to capture aspects of social attention that may arise only in authentic social contexts (or at least be difficult to detect in nonsocial contexts; e.g., Kingstone, 2009; Risko et al., 2016).

The above considerations have led to the development of the dual function perspective in social attention research, wherein social attentional behavior (e.g., an overt shift of attention) is viewed both as a means of collecting social information and a channel through which information is communicated to others (Gobel, Kim, & Richardson, 2015; Risko et al., 2016; see also Argyle & Cook, 1976). Through directing one’s overt attention to social agents, individuals gain information (e.g., emotional state) and communicate information to them (e.g., interest; Kleinke, 1986), whether intentionally (e.g., a signal) or not (Wu, Bischof, & Kingstone, 2013, 2014). The existence of these two functions provides one potential explanation for inconsistencies between social attentional behavior expressed in less authentic contexts and social attentional behavior expressed in authentic social contexts. That is, social attention research employing schematic faces and images of social agents may not be tapping into both of these functions (or at least not to the same extent). For example, although looking at a person in an image of a social scene could reveal the information valued by the looker, the costs/benefits of the potential information communicated by this behavior (i.e., gazing at the person in the scene) need not be considered. In other words, traditional research relying on “nonsocial” social stimuli has likely revealed regularities in social attentional behavior relevant to information acquisition, but expecting such regularities to emerge in authentic social contexts that require considering the communicative consequences of the distribution of one’s attention might be a bridge too far.

The notion that where we attend (overtly) communicates information, and that we monitor this information, has some empirical support (Emery, 2000; Kleinke, 1986; Patterson et al., 2002; Risko et al., 2016; Wu et al., 2014). For example, research on “civil inattention” has demonstrated that individuals will look away from a social agent when in an authentic social context to show respect for the other social agent’s privacy (Goffman, 1963; Patterson et al., 2002; Zuckerman, Miserandino, & Bernieri, 1983). More directly, Risko and Kingstone (2011; see also Nasiopoulos, Risko, Foulsham, & Kingstone, 2015) had participants sit in a room with a provocative stimulus (i.e., a swimsuit calendar), and participants were either aware or not that their gaze was being monitored (e.g., by a mobile eye tracker). Critically, participants changed their looking behavior as a function of whether or not they thought their gaze was being monitored, looking much less at the provocative stimulus when they believed that their eyes were being monitored (i.e., their eyes could communicate information about themselves to others). These results are consistent with individuals monitoring what their gaze may communicate to other social agents and modifying their looking behavior accordingly.

### Dual functions and cognitive load

When considering social attention from a dual function perspective, the need exists to manage potential competition between the desire to acquire information and the need to monitor what is being communicated (e.g., a desire to look at someone may conflict with the desire to not stare). Managing competition or processing conflict is thought to be a function of a limited capacity executive control system (Miyake et al., 2000; Spagna, Mackie, & Fan, 2015). For example, taxing the cognitive system has been shown to interfere with selective attention in a typical flanker task (Lavie, Hirst, De Fockert, & Viding, 2004). Lavie et al. (2004) argued that under a higher cognitive load, the participant’s available control resources were reduced, and as such, increased processing of distractor information occurred. Thus, higher load decreases our ability to manage competing processes. Taking both of these ideas together – (i) that the dual functions of gaze could compete and (ii) the system responsible for managing competition is capacity limited – suggests that social attention, at least in authentic social contexts, may be sensitive to variations in cognitive load. We examine this general hypothesis in the present investigation.

Limited work has been done on the influence of manipulations of cognitive load on social attention. In a recent notable exception, Pecchinenda and Petrucci (2016) examined the role of cognitive load in gaze cueing using emotional faces. They found that under higher cognitive load, greater interference occurred from angry emotional distractors, suggesting that the load compromised control processes, thereby making it harder to reduce interference from emotional information.

In other research where cognitive load was not directly manipulated but gaze was examined in face-to-face interactions, researchers have found that both adults and children will avert their gaze when completing a cognitively demanding task (Doherty-Sneddon, Bruce, Bonner, Longbotham, & Doyle, 2002; Glenberg, Schroeder, & Robertson, 1998). For example, Doherty-Sneddon et al. (2002) investigated 8-year-old children’s gaze behavior with respect to the experimenter while completing arithmetic and verbal tasks. They found that the more challenging the cognitive task (i.e., the more cognitively demanding the task was) the less frequently the children would gaze at the experimenter sitting in front of them. This behavior might reflect in part an eye-contact effect, wherein making direct eye contact represents an attentional liability best avoided under demanding conditions (e.g., Senju & Johnson, 2009).

Additional indirect evidence for an influence of cognitive load on social attentional behavior can be gleaned from research examining the impact of cell phone use on individual’s attention. Talking on a cell phone introduces a form of cognitive load (i.e., consuming attentional resources; Strayer & Johnston, 2001; Walker, Lanthier, Risko, & Kinstone, 2012); thus, how individuals look at other social agents while talking on a cell phone can provide some clues as to how cognitive load might influence social looking. For example, Patterson, Lammers, and Tubbs (2014) examined the influence of cell phone use on social looking behaviors in an authentic social context and found that participants using cell phones looked toward the oncoming pedestrian but failed to react to them (e.g., smile or nod), which the authors suggest was due to the cell phone use reducing attentional resources. Additionally, cell phone users have also been shown to exhibit inattentional blindness to other social agents. In a design similar to the pedestrian passing scenario, Hyman, Boss, Wise, McKenzie, and Caggiano (2010) observed individuals talking on cellphones, using personal music devices, and using no technology as they passed through a town square. Within the square, they had a unicycling clown cycle around a large sculpture. Hyman et al. (2010) found that only 25% of those talking on cell phones reported noticing the clown, compared to those with personal music devices and those alone, 61% and 51%, respectively (Hyman et al., 2010). Although both of these studies suggest that cognitive load can modulate social looking, neither experimentally manipulated load nor had a direct measure of gaze (i.e., eye tracking). In the present investigation, we directly manipulate cognitive load in an authentic pedestrian crossing paradigm (similar to Patterson et al., 2014) while individuals’ eye movements were monitored via a mobile eye-tracker.

### Mobile eye-tracking

According to the dual function of gaze framework, investigating social attention in authentic social contexts is important. While seemingly intuitive, it seems clear that the rapid growth of the social attention field has not prioritized this necessity. Arguably, this omission reflects technological challenges, as it is difficult to measure attentional behavior in great detail in authentic social contexts. Mobile eye-tracking technology provides a potential solution to this challenge by providing the opportunity to measure gaze behavior without restricting the stimulus to a computer screen (as with standard desktop eye trackers; Tatler & Land, 2016). Nevertheless, significant hurdles to using mobile eye-trackers remain (e.g., coding gaze locations is done by hand; implicit social presence created by the mobile eye tracker; Nasiopoulos et al., 2015; Risko & Kingstone, 2011), which likely contributes to the fact that most work using them has featured small sample sizes (e.g., Land, Mennie, & Rusted, 1999; Scrafton, Stainer, & Tatler, 2017). All of the reported experiments here take advantage of this technology and feature larger samples relative to other mobile eye tracking studies mentioned above, which have samples of 10 people or less.

### The present investigation

In our first two experiments, participants completed a short walk around a building while wearing a head-mounted mobile eye tracker. All participants on a specific hallway were passed by a confederate, Experiment 1 had a between-subject manipulation, and Experiment 2 had a within-subject manipulation. Critically, during the walk, we manipulated cognitive load using an auditory 2-back task (see Jaeggi, Buschkuehl, Perrig, & Meier, 2010). That is, in the load condition, participants completed the entire walk while also performing an auditory 2-back (presented via headphones), and in the no-load condition participants completed the entire walk while simply listening to the auditory 2-back stimuli. If social attentional behavior, measured by looks toward the pedestrian, in a typical pedestrian crossing context draws on limited capacity resources, then looking behavior toward the pedestrian should be modulated by variation in cognitive load (i.e., load vs. no-load). The direction of such an effect would provide novel insight as to how such resources are naturally being allocated. In Experiment 3, participants performed the same task but instead of actually walking through a hallway and encountering live social agents, they viewed a video (life-size) of such a walk. This provides a critical contrast between social attentional behavior in an authentic social context versus a more typical attempt at a simulation of one. Thus, across the three experiments, we could examine the influence of cognitive load on social attentional behavior both in a more and less authentic social context.

While not our initial focus, the present design also allowed for an examination of how proximity influences social attentional behavior. Foulsham, Walker, and Kingstone (2011) reported a reduction in gaze toward a passing social agent as that agent came closer to the participant. Interestingly, this pattern was more pronounced when the participant was in a social situation (i.e., walking by another person) versus when the participant was watching a video of that same situation. The present investigation allows for an examination of the extent to which load moderates these patterns. We decided to include proximity into our analysis since this feature is informative theoretically with respect to the mechanism underlying these effects. For example, if cognitive load interacts with proximity, this interaction would suggest that the looking behavior toward a confederate either relies on limited capacity resources (i.e., if there was a reduction in that effect) or in some way reflects a strategy to conserve resources (i.e., if there was an increase in the magnitude of that effect).

## Experiment 1

### Methods

#### Participants

A total of 104 undergraduate students from the University of Waterloo completed this study for course credit. Participants were randomly assigned to either the no-load condition or the load condition. Twenty-four participants were excluded for one of the following reasons: three of the participants got lost during the walk, two were excluded due to confederate error, two encountered another person in the target hallway, and 17 had technical issues (e.g., calibration error, video recording failure, or N-back audio failure). The distribution of the remaining 80 participants was 42 in the no-load condition and 38 in the load condition. No demographic data were collected. We conducted a power analysis for the number of participants needed to achieve a minimum .80 power (alpha = .05) for the main effect of cognitive load condition (i.e., a two-tailed independent t-test), with the assumption that we would have a medium-large effect size (i.e., Cohen’s *d* = 0.65) for both of our dependent variables. With these criteria we needed a minimum of 39 participants in each group.

#### Stimuli

**Fig. 1:**
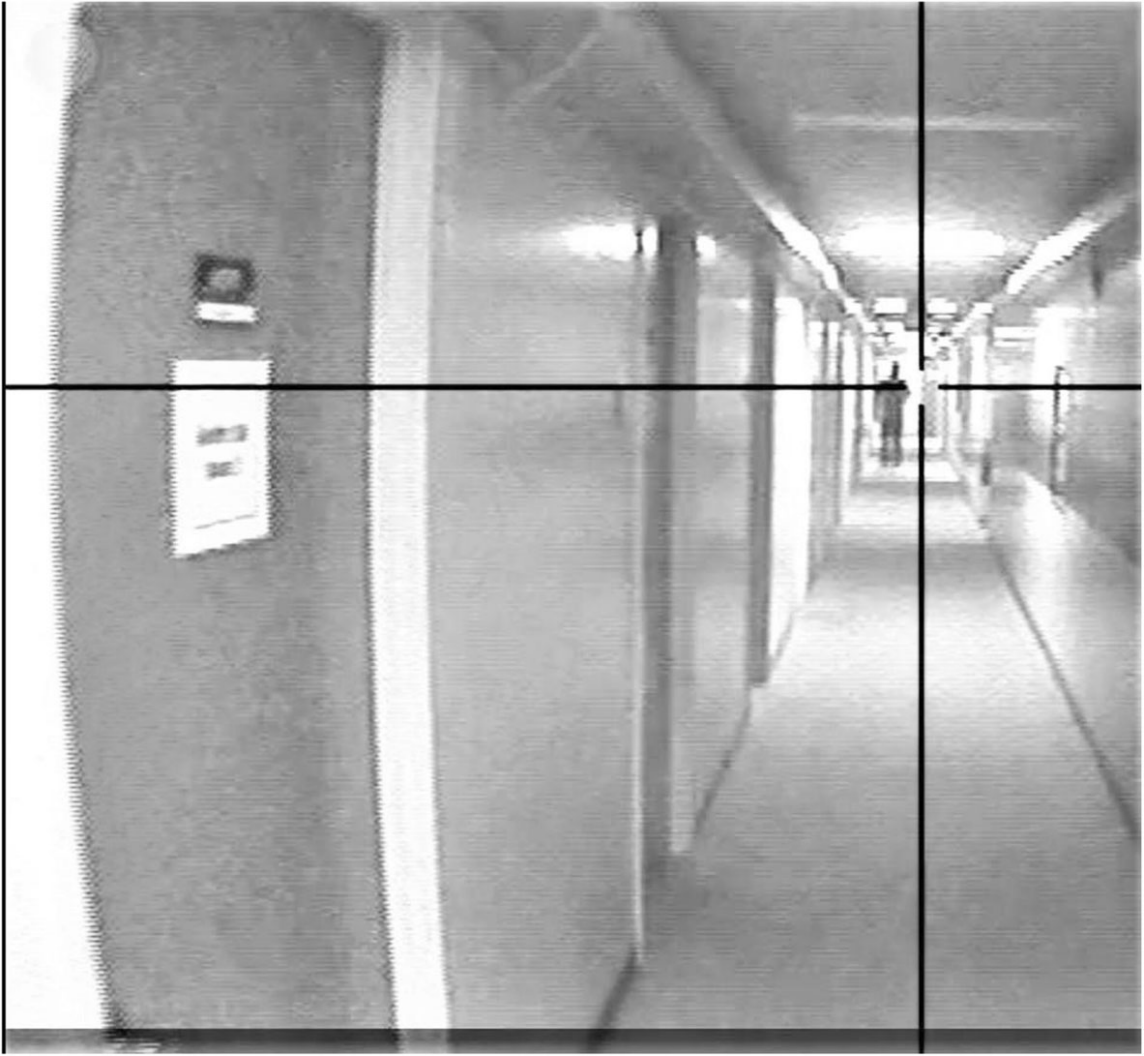
Screen capture from a video of the hallway walk from the mobile eye tracker. The confederate is at the end of the hallway looking toward his or her own phone. The cross represents where the fixation was located. This was the start of the hallway. Seven doors are located in this hallway: five on the left and two on the right at the end of the hallway

Participants walked down four hallways (a rectangular shape) in the psychology building on campus. This building mainly consists of a series of almost identical hallways with many small offices (see Fig. 1). The full walk was approximately 300 m, with the target hallway (i.e., the hallway where the participant passed the confederate) being approximately 112 m in length. When participants began walking down the third of the four hallways (i.e., the target hallway) the confederate would be in view, walking toward the participant at the opposite end of the hallway. A subset of participants (*n* = 52) also encountered a different confederate sitting in a different hallway due to a secondary manipulation we do not report here. The presence of this additional confederate did not change the results reported below for fixation behavior qualitatively, (all *p*’s > .05, largest BF_inc_ = 0.68, see supplemental material). The confederate on the target hallway could have been male or female and was dressed casually. The gender of the confederate was not intentionally manipulated but instead reflected the availability of the confederates. In total, 54 participants saw a male confederate, and 26 participants saw a female confederate. These confederates were distributed across the no-load (28 male, 13 female) and the load (26 male, 13 female) conditions, and participants saw one of 10 confederates. The confederate would walk past the participant without gazing toward them; the confederates were always looking down, pretending to be occupied with their phone.

The N-back task used in the load condition was a 2-back auditory task with letter stimuli. When participants heard the same letter repeated from two letters before, they needed to press a button on a mouse they were holding in their hand. Five letters were used: B, F, H, J, and L. The letters were presented for 500 ms with a 1500 ms break between each letter for response. The letters were presented randomly with replacement; thus, a 1 in 5 chance existed that the next randomly generated letter would be a target. The number of targets varied by participant because the time to complete the walking task varied by participant. Participants in the load condition wore headphones and responded to the target letters using a wireless mouse. Those in the no-load condition also wore the headphones, but they just listened to the stream of letters.

At the end of the experiment the participants completed the Social Desirability Scale (SDS). This questionnaire consists of 33 true and false questions and is used to assess how much an individual is concerned with social approval (Crowne & Marlowe, 1960). For example, one question asks “I am always careful about my manner of dress” true or false? This scale has a reliability of .88 (Crowne & Marlowe, 1960). We do not report analyses of these data here (see supplemental material).

#### Apparatus

Participants wore a Positive Science Mobile Eye Tracker. The eye tracker has two components: a headset and a backpack. Participants wore both for the duration of the walk (approx. 5 min). The headset resembles the frames of a pair of glasses, and is lightweight. It contains two cameras: one to record the eye and one to record the scene of the hallway participants are looking at. The backpack, which weighs 2.2 kg, was worn by participants and contained the laptop that ran the software for the eye tracker, as well as two batteries for the system. The scene video and eye sampling rate were 30 frames per second. While the video was recorded for the entire walk, for the purpose of the study, only the video from the target hallway was analyzed. In addition to the headset and backpack, participants wore headphones so that they could hear the letters for the N-back task, and they carried a wireless mouse to respond to the N-back target, even if they were in the no-load condition where no response was required.

#### Procedure

Participants entered the lab and gave written consent before the experiment began. Participants were then fitted with the mobile eye tracker and headphones for the N-back task. The mobile eye tracker was then calibrated. Calibration required participants to fixate on a pen the experimenter was holding approximately 1 m away and then to follow it with their gaze as the experimenter moved the pen in an X shape. This ensured the eye tracker was correctly detecting fixations and mapping their location to the scene footage. The calibration was completed using Yarbus eye-tracking software (information on the software can be found through the positive science website: http://positivescience.com/software/). Once calibration was complete, participants began the experiment. The experimenter explained that they simply needed to walk the required route (the four hallways) leading them back to the testing room while wearing the eye tracker, they were not given any more detail about the purpose of the study. If they were in the load condition they were told to complete the N-back task while walking this route, or else just listen to the audio. Once they returned, their eye tracker was removed, they completed the social desirability scale, and then they were debriefed.

### Results

Raw video files of the eye and scene were recorded using the Positive Science live capture software. These two videos were then synced and merged together using Yarbus eye-tracking software. The software merged video was then analyzed through the GazeTag software application to identify all fixations made by the participant (see a demonstration here: http://positivescience.com/preview/GT_shortForWeb/GT_shortForWeb.html). A fixation was defined as gaze remaining in one location (within 10 pixels) for more than 100 ms (the videos were recorded with a 640 × 480 pixel format). The software extracts the scene image (i.e., the hallway) associated with each fixation. These scenes with the fixation location overlaid were then used to code fixations on a frame-by-frame basis as located on the confederate’s head, the confederate’s body, proximal to the confederate (this was a judgement made by the coder, a fixation was coded as proximal if the fixation was in the direction of the confederate but did not land on the confederate), and off the confederate (i.e., everything else). The latter two categories were combined for analysis. In addition, the target hallway was divided into “near” and “far” from the confederate defined temporally such that the participant was considered near when they were within 3 s of reaching the confederate and far otherwise. Time was used rather than distance because it was challenging to determine how far the confederate was through the video software. To help with visualization of the near condition (i.e., within 3 s of passing the confederate), participants were likely no further than 10 m away from the confederate.

Below we first report descriptors for the N-back task and overall timing of the hallway walk (walking time, time in view). The latter measures are provided for descriptive purposes. We then report analyses of the proportion of fixations directed toward the confederate (i.e., the number of fixations that landed on the confederate divided by the total number of fixations) and proportion of time (i.e., the amount of time divided by the time the confederate was in view) as a function of proximity (near, far) and condition (no-load, load). Lastly, we report an analysis focused on fixations toward the confederate’s head or body. While we focus on null-hypothesis testing when interpreting results, Bayes factors were calculated for each effect using JASP statistical software (Love et al., 2015). Given that interaction effects are always produced within a model with main effects, we have chosen to report the Bayes factor inclusion values for our ANOVAs (reported as BF_inc_ throughout the paper), which compare the specific effect to matched models (i.e., other models with the effect) to determine the strength of the evidence for that distinct effect. For Bayes factors inclusion, the prior inclusion probability is calculated as the sum of the prior probabilities of all models that include that effect (JASP Team, 2020). Bayes factors for the t-test use the software’s default prior of .71 (or √2/2), which represents a Cauchy distribution (Rouder, Speckman, Sun, & Morey, 2009). Bayes factors between 1 and 3 should be interpreted as anecdotal evidence, 3–10 as substantial evidence, and factors of 10+ as strong evidence (Jarosz & Wiley, 2014).

#### Non-eye movement measures

##### N-back accuracy

N-back accuracy was calculated as the proportion of true positive, false positive, true negative, and false negative responses (see Table 1). N-back data were missing for one of the participants in Experiment 1. While limited analyses could be conducted on the N-back data, the task was challenging (i.e., participants were not at ceiling), and at the same time, participants were putting effort into completing it (i.e., the d’ score suggests they are performing above chance).

**Table 1:** Proportion of responses for the N-back task across Experiments 1–3

	True positive	False positive	True negative	False negative	d’
	Mean (95%CI)	Mean (95%CI)	Mean (95%CI)	Mean (95%CI)	
**Experiment 1**	0.44 (0.34, 0.54)	0.11 (0.07, 0.15)	0.90 (0.86, 0.94)	0.55 (0.45, 0.65)	1.03
**Experiment 2**	0.53 (0.41, 0.64)	0.12 (0.09, 0.16)	0.88 (0.84,0.91)	0.47 (0.36, 0.59)	1.17
**Experiment 3**	0.59 (0.52, 0.67)	0.10 (0.07, 0.13)	0.91 (0.88,0.94)	0.39 (0.31, 0.47)	1.55

##### Walking time

Those in the no-load condition (28.92 s) took significantly less time to walk down the target hallway compared to those in the load condition (31.92 s), *t*(78) = 2.25, *p* = .03, *d* = 0.50, BF_10_ = 2.01.

##### Time in view

Participants in the no-load condition took on average 9.71 s to pass the confederate, compared to the load condition, which averaged 11.14 s, and this difference was not significant, *t*(78) = 1.72, *p* = .09, *d* = 0.39, BF_10_ = 0.84.

#### Eye movement measures – on vs. off confederate

##### Fixations

In Table 2 we provide basic descriptive statistics for the proportion of fixations, proportion of time, number of fixations, and total time for fixations on the confederate. Given that the near and far categorizations of fixations are nonequivalent temporally and that the walking time differs across conditions, we focus analyses on proportional measures, specifically, the proportion of fixations on the confederate and the proportion of time spent looking at the confederate. That said, the patterns of means in terms of nonproportional measures are qualitatively similar.

**Table 2:** Fixation behavior toward the confederate by Condition (no-load, load) and Proximity (near, far) in Experiment 1; time is reported in seconds

	Near	Far
	Mean (95%CI)	Mean (95%CI)
***Proportion of fixations***		
No-load	0.24 (0.19, 0.30)	0.31 (0.24, 0.37)
Load	0.12 (0.05, 0.19)	0.20 (0.13, 0.26)
***Proportion of time***		
No-load	0.25 (0.18, 0.32)	0.33 (0.26, 0.41)
Load	0.11 (0.04, 0.18)	0.22 (0.15, 0.30)
***Number of fixations on the confederate***		
No-load	1.88 (1.44, 2.32)	5.29 (3.82, 6.75)
Load	0.89 (0.36, 1.43)	3.58 (2.38, 4.77)
***Total time fixating on the confederate***		
No-load	0.45 (0.33, 0.57)	1.63 (1.13, 2.13)
Load	0.22 (0.07, 0.36)	1.06 (0.64, 1.48)
***Total fixations***		
No-load	7.83 (7.14, 8.53)	17.40 (14.43, 20.38)
Load	6.84 (6.04, 7.65)	19.42 (16.34, 22.50)
***Total time***		
No-load	1.80 (1.63, 1.96)	4.60 (3.86, 5.34)
Load	1.79 (1.60, 1.98)	4.89 (4.01, 5.76)

##### Proportion of fixations

**Fig. 2:**
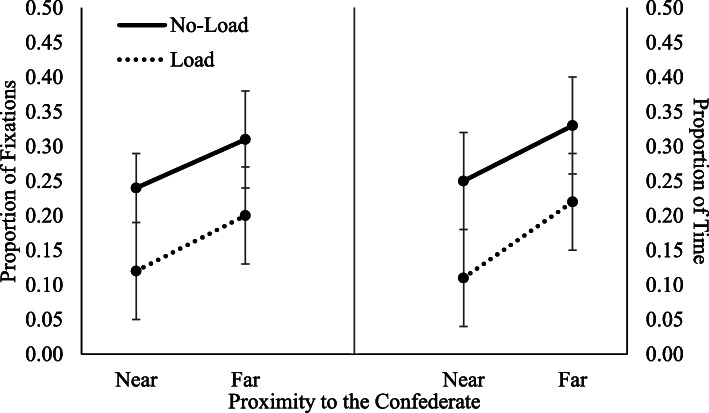
The mean proportion of fixations (left panel) and time spent on the confederate (right panel) when the confederate was in view for Condition (no-load, load) and Proximity (near, far) in Experiment 1. Bars show 95% confidence intervals

We conducted a Condition (no-load, load) by Proximity (near, far) mixed ANOVA on the proportion of fixations directed toward the confederate. A significant main effect of Condition, *F*(1, 78) = 12.05*, p* < .001, ${\eta}_G^2$ =.08, BF_inc_ = 20.79, was identified, such that the no-load condition had a higher proportion of fixations on the confederate (*M* = .27) compared to the load condition (*M* = .16). In addition, a significant main effect of Proximity, *F*(1, 78) = 5.26*, p* = .03, ${\eta}_{\mathrm{G}}^2$ =.03, BF_inc_ = 2.33, was identified, such that when near, the proportion of fixations on the confederate (*M* = .18) was lower compared to when far (*M* = .25). No interaction was observed between Condition and Proximity, *F*(1, 78) = 0.05, *p = .*83, ${\eta}_G^2$ <.01, BF_inc_ = 0.24 (see Fig. 2). Further analyses revealed no main effect of confederate gender or of interactions including confederate gender.

##### Proportion of time

We found the same pattern of results when we looked at the proportion of time fixating on the confederate. A significant main effect of Condition, *F(*1, 78) = 10.84*, p* = .001, ${\eta}_G^2$ =.08, BF_inc_ = 23.81, was observed, such that those in the no-load condition spent a higher proportion of time looking at the confederate (*M* = .29) compared to those in the load condition (*M* = .17). As well, a significant main effect of Proximity, *F(*1, 78) = 10.18*, p* = .002, ${\eta}_G^2$ =.05, BF_inc_ = 21.88, was observed, such that when near, participants spent proportionally less time looking at the confederate (*M* = .18) than when they were far (*M* = .28). No interaction was observed between Condition and Proximity, *F*(1, 78) = 0.22, *p* = .64, ${\eta}_G^2$ <.01, BF_inc_ = 0.19 (see Fig. 2), and no effects were observed with confederate gender.

##### Eye movement measures – head vs. body

In the next analysis, we compared fixation locations within the confederate. The fixations toward the confederate were divided into those that were located on the confederate’s head vs. on the confederate’s body (see Table 3).

**Table 3:** Proportion of fixations toward the head and body by Condition (no-load, load) and Proximity (near, far) in Experiment 1

	Head		Body	
	Near	Far	Near	Far
	Mean (95%CI)	Mean (95%CI)	Mean (95%CI)	Mean (95%CI)
***Proportion of fixations***				
No-load	0.15 (0.10, 0.20)	0.11 (0.07, 0.15)	0.09 (0.06, 0.13)	0.20 (0.14, 0.25)
Load	0.05 (0.01, 0.09)	0.07 (0.03, 0.10)	0.07 (0.02, 0.12)	0.13 (0.07, 0.19)
***Proportion of time***				
No-load	0.17 (0.11, 0.23)	0.15 (0.09, 0.20)	0.08 (0.05, 0.11)	0.19 (0.13, 0.25)
Load	0.05 (0.01, 0.09)	0.06 (0.03, 0.10)	0.06 (0.01, 0.11)	0.16 (0.09, 0.22)

##### Proportion of fixations

We conducted a Condition by Proximity by Location (head, body) mixed ANOVA on the proportion of fixations toward the confederate. No significant main effect of Location, *F*(1,78) = 2.26, *p* = .14, ${\eta}_G^2$ =.01, BF_inc_ = 0.49, and no significant Condition by Location interaction, *F*(1,78) = 0.41, *p* = .52, ${\eta}_G^2$ <.01, BF_inc_ = 0.20, were observed. A significant Proximity by Location interaction, *F*(1,78) = 11.39, *p* = .001, ${\eta}_G^2$ =.02. BF_inc_ = 22.10, was observed, such that there was no difference between the near (*M* = 0.10) and far condition (*M =* 0.08) for fixations toward the head, *t*(79) = 0.78, *p* = .44, *d* = 0.09, BF_10_ = 0.17; however, for fixations toward the body, significantly fewer fixations were observed when near the confederate (*M* = 0.09) compared to when far (*M* = 0.16), *t*(79) = 3.68, *p* < .001, *d* = 0.41, BF_10_ = 52.21. No significant 3-way interaction, *F*(1,78) = 2.47, *p* = .12, ${\eta}_G^2$ <.01, BF_inc_ = 0.65, was observed.

##### Proportion of time

The same pattern of results was found for the proportion of time. No main effect of Location, *F*(1, 78) = 0.47*, p* = .50, ${\eta}_G^2$ <.01, BF_inc_ = 0.15, was observed, nor a significant interaction between Condition and Location, *F*(1, 78) = 3.51, *p* = .07, ${\eta}_G^2$ =.01, BF_inc_ = 2.01. A significant Proximity by Location interaction, *F*(1, 78) = 13.48*, p* < .001, ${\eta}_G^2$ =.03, BF_inc_ = 21.31, was observed, such that the proportion of time spent fixating on the confederate’s head did not significantly differ based on proximity (near *M* = 0.11, far *M* = 0.07), *t*(79) = 0.33, *p* = .74, *d* = 0.04, BF_10_ = 0.13, but participants spent a lower proportion of time fixating on the confederate’s body when near (*M* = 0.07) compared to when far (*M* = 0.17), *t*(79) = 4.70, *p* < .001, *d* = 0.53, BF_10_ = 1540.62. No significant three-way interaction, *F(*1,78) = 0.78*, p* = .38, ${\eta}_G^2$ <.01, BF_inc_ = 0.32, was observed.

### Discussion

In Experiment 1, a significant effect of cognitive load on looking behavior was observed in an authentic pedestrian passing scenario, suggesting that cognitive load modulates social attentional behavior. Specifically, those in the load condition fixated less often on the passing confederate and spent less time fixating on the confederate compared to those in the no-load condition. With respect to proximity, across conditions, participants exhibited a higher proportion of fixations and proportion of time spent fixating on the confederate when they were far, compared to when they were near. This effect of proximity is similar to that observed by Foulsham et al. (2011). Load did not modulate this pattern. In addition, this effect of proximity appeared to be largely a product of a reduction in looks toward the confederate’s body as the confederate drew nearer (i.e., looks toward the head were equivalent in the near and far conditions). That is, a significant interaction was observed between proximity (near, far) and location (head, body). The Bayes factors associated with these results were consistent with the conclusions drawn.

## Experiment 2

In Experiment 2 we sought a replication of Experiment 1. In addition, we moved to a within-participant manipulation of load. A within-participant design should increase our power to detect more subtle effects (e.g., the influence of cognitive load on looks within the person) and reduce concerns that the results from Experiment 1 reflected chance assignment of, for example, socially anxious individuals to the load group, thus explaining the reduction in gaze toward the confederate (e.g., Hessels, Holleman, Cornelissen, Hooge, & Kemner, 2018; Langer & Rodebaugh, 2013). Thus, participants walked two routes instead of one: one in the load condition and one in the no-load condition.

### Methods

#### Participants

A total of 69 undergraduate students from the University of Waterloo completed this study for course credit. Nine participants were removed because they got lost during the walking route (seven in the no-load condition, and two in the load condition), two were removed due to confederate error, 11 were removed for technical issues (e.g., video recording error, N-back audio failure, or calibration issue), and one was removed because there was another person who appeared on the walking route blocking the confederate; 46 participants remained. No demographic data were collected. We aimed to collect 48 participants. This gave us .80 power with an alpha = .05 to detect a minimum effect size of approximately *d* = .40 for the main effect of load. The effect of load on proportion of fixations was *d* = .78 in Experiment 1.

#### Stimuli

The stimuli were the same as Experiment 1 with the following exceptions. All participants completed two routes – one in each condition. Both of the routes consisted of four hallways in a rectangular shape as in Experiment 1. One of the walking routes was the same four hallways used in Experiment 1; the other set of four hallways was a new path around the floor. The two paths were matched for distance, and both had the same walking distance as in Experiment 1. As such, the target hallway with the confederate was approximately 113-m long, with a total walking path of approximately 300 m. The two routes were counterbalanced for which route the participant took first as well as for which task condition they completed while walking (N-back, no N-back). Each participant saw two confederates (one for each walking route). Once again, the confederates could have been male or female depending on availability, and they did not make eye contact with the participant; however, unlike in Experiment 1 they were not all looking down toward their phone. Confederates were instructed to do what felt natural while not making eye contact, which led to 58 of the confederates pretending to be occupied by a cell phone (26 in the no-load condition and 32 in the load), and the other 34 simply walked past while looking down or away. Analyses revealed no effect of confederate behavior (i.e., phone vs no phone) on the participants’ looking behavior (all *p’s* > .05, largest BF_inc_ = 0.69 see supplemental for means), and as such, the reported analyzes collapse across this factor. In total, 45 of the confederates seen by participants were male confederates and 47 of the confederates seen were female confederates. These confederates were distributed across the no-load (19 male, 27 female) and the load (26 male, 20 female) conditions, and participants saw only one of the possible confederates for each of their walking routes. The make-up of the confederate pairs were 11 male-male, 12 female-female, and 23 mixed male-female.

#### Apparatus

The apparatus used was the same as in Experiment 1. Since participants completed both the load and no-load conditions, the time spent wearing the headset, backpack, and headphone was doubled (approx. 10 min).

#### Procedure

Participants came into the laboratory, gave consent and were calibrated with the eye tracker in the same manner as in Experiment 1. Once calibrated, participants were told whether they were to complete the load or the no-load condition and were then told the four-hallway route to walk. Once they returned, they were told to complete the next condition, and then instructed to walk down a different set of four hallways (still leading back to the same testing room). Once they completed the second walk, the eye tracker was removed, they completed the social desirability scale, and were debriefed.

### Results

Data were coded in the same manner as Experiment 1. Although this experiment uses a within-subject design, all Cohen’s d values reported are calculated as between subject to make it easier to compare to Experiment 1. As such, they are more conservative.

#### Non-eye movement measures

##### N-back accuracy

Given that our N-back was randomly generated, five participants were excluded from the N-back analysis because they never received a target letter. N-back accuracy was calculated as the proportion of true positive, false positive, true negative, and false negative responses (see Table 1).

##### Walking time

As in Experiment 1, while performing the no-load task, participants took significantly less time (*M =* 28.54 s) to walk the target hallway compared to when they completed the load task (*M =* 30.39 s), *t*(45) = 3.65, *p* < .001, *d* = 0.45, BF_10_ = 40.79.

##### Time in view

When participants were in the no-load condition they took, on average, 10.88 s to pass the confederate, compared to when in the load condition, which averaged 11.49 s. This difference was not significant, *t*(45) = 1.03, *p* = .31, *d* = 0.24, BF_10_ = 0.26.

#### Eye movement measures – on vs. off confederate

##### Fixations

The proportion of fixations, proportion of time, number of fixations and total time were recorded for fixation behavior toward the confederate (see Table 4). No effect of order (no-load, load) or which hallway was walked down first, was observed for any of the fixation variables (all *p*’s > .05).

**Table 4:** Fixation behavior toward the confederate by Condition (no-load, load) and Proximity (near, far) in Experiment 2; time is reported in seconds

	Near	Far
	Mean (95%CI)	Mean (95%CI)
***Proportion of fixations***		
No-load	0.31 (0.24, 0.39)	0.26 (0.20, 0.33)
Load	0.15 (0.10, 0.21)	0.25 (0.18, 0.31)
***Proportion of time***		
No-load	0.34 (0.25, 0.42)	0.29 (0.22, 0.37)
Load	0.12 (0.07, 0.17)	0.29 (0.20, 0.37)
***Number of fixations on the confederate***		
No-load	2.70 (1.97, 3.42)	4.57 (3.42, 5.71)
Load	1.13 (0.73, 1.53)	4.72 (3.25, 6.19)
***Time fixating on the confederate***		
No-load	0.62 (0.45, 0.80)	1.56 (1.06, 2.07)
Load	0.22 (0.11, 0.34)	1.63 (1.08, 2.17)
***Total fixations***		
No-load	8.07 (7.34, 8.79)	18.93 (16.47, 21.40)
Load	7.24 (6.47, 8.00)	21.11 (17.53, 24.69)
***Total time***		
No-load	1.83 (1.66, 2.00)	5.27 (4.38, 6.16)
Load	1.76 (1.56, 1.95)	5.47 (4.50, 6.43)

##### Proportion of fixations

**Fig. 3:**
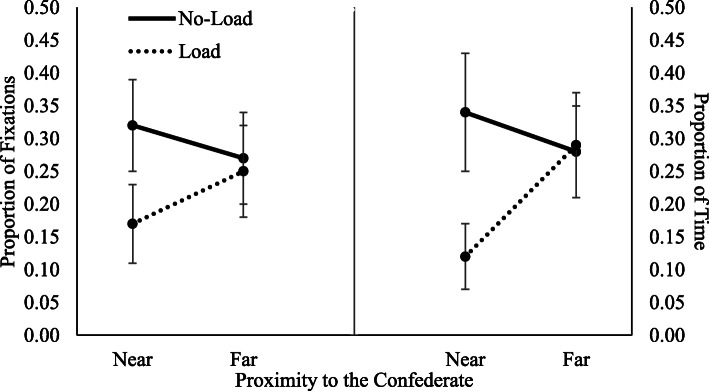
The mean proportion of fixations (left panel) and time spent on the confederate (right panel) when the confederate was in view for Condition (no-load, load) and Proximity (near, far) in Experiment 2. Bars show 95% confidence intervals

A Condition by Proximity repeated measures ANOVA was conducted on the proportion of fixations directed toward the confederate. As with Experiment 1, a significant main effect of Condition, *F*(1, 45) = 7.69, *p* = .008, ${\eta}_G^2$ =.04, BF_inc_ = 8.62, was observed, such that in the no-load condition there was a higher proportion of fixations toward the confederate (*M =* 0.29) compared to the load condition (*M* = 0.20). No significant main effect of Proximity, *F*(1, 45) = 0.39, *p =* .54, ${\eta}_G^2$ <.01, BF_inc_ = 0.17, was observed. Also, a significant interaction between Condition and Proximity, *F*(1, 45) = 9.54, *p* = .003, ${\eta}_G^2$ =.03, BF_inc_ = 3.03, was observed. As shown in Fig. 3, this reflects a larger effect of condition when near (no-load *M* = 0.31, load *M* = 0.15), *t*(45) = 3.61, *p* < .001, *d* = 0.73, BF_10_ = 37.44, than when far (no-load *M* = 0.26, load *M* = 0.25), *t*(45) = 0.46, *p* = .65, *d* = 0.07, BF_10_ = 0.18. Further analyses revealed no main effect of the confederate’s gender or of interactions including gender of the confederate.

##### Proportion of time

Similar to the proportion of fixations, when we looked at the proportion of time fixating on the confederate, a significant main effect of condition, *F*(1, 45) = 9.73, *p* = .003, ${\eta}_G^2$ =.05, BF_inc_ = 13.73, was observed, such that, when in the no-load condition, participants spent a significantly higher proportion of time fixating on the confederate (*M* = 0.32) compared to when in the load condition (*M* = 0.20). No significant main effect of Proximity, *F*(1, 45) = 2.93, *p* = .09, ${\eta}_G^2$ =.01, BF_inc_ = 0.50, was observed. Also a significant interaction between Condition and Proximity, *F*(1, 45) = 15.00, *p* < .001, ${\eta}_G^2$ =.04, BF_inc_ = 17.25, was observed, such that, when participants were near, the proportion of time spent looking at the confederate was higher in the no-load condition (*M* = .34) compared to the load condition (*M =* .12), *t*(45) = 4.50, *p* < .001*, d* = 0.92, BF_10_ = 340.02, but no significant difference was observed between the no-load (*M* = .29) and the load conditions (M = .29) when far, *t*(45) = 0.12, *p =* .91, *d =* 0.02, BF_10_ = 0.16 (see Fig. 3). Further analyses revealed no main effect of confederate gender or interactions including confederate gender.

#### Eye movement measures – head vs. body

##### Proportion of fixations

The fixations toward the confederate were divided into specific locations: head and body (see Table 5). We conducted a Condition by Proximity by Location repeated measures ANOVA on the proportion of fixations toward the confederate. No main effect of Location, *F*(1, 45) = 2.07, *p* = .16, ${\eta}_G^2$ <.01, BF_inc_ = 0.92, and no interaction between Condition and Location, *F*(1, 45) = 3.21, *p* = .08, ${\eta}_G^2$ <.01, BF_inc_ = 1.37, were observed. A significant interaction between Proximity and Location, *F*(1, 45) = 13.40, *p* < .001, ${\eta}_G^2$ =.03, BF_inc_ = 14.81, was observed, such that the proportion of fixations toward the head was higher near (*M* = 0.13) compared to when far (*M =* 0.08), *t*(45) = 2.16, *p* = .04, *d* = 0.40, BF_10_ = 1.30, and the opposite was the case for the proportion of fixations toward the body, near (*M =* 0.10) compared to far (*M =* 0.17), *t*(45) = 2.72, *p* = .009, *d* = 0.48, BF_10_ = 4.15. No significant three-way interaction, *F*(1, 45) = 3.04, *p* = .09, ${\eta}_G^2$ <.01, BF_inc_ = 0.75, was observed.

**Table 5:** Proportion of fixations toward the head and body for Condition (no-load, load) and Proximity (near, far) in Experiment 2

	Head		Body	
	Near	Far	Near	Far
	Mean (95%CI)	Mean (95%CI)	Mean (95%CI)	Mean (95%CI)
***Proportion of Fixations***				
No-load	0.20 (0.14, 0.26)	0.09 (0.05, 0.12)	0.12 (0.07, 0.16)	0.17 (0.11, 0.24)
Load	0.06 (0.03,0.09)	0.08 (0.04, 0.12)	0.09 (0.05, 0.14)	0.17 (0.11, 0.23)
***Proportion of Time***				
No-load	0.21 (0.14, 0.28)	0.11 (0.06, 0.15)	0.13 (0.07, 0.18)	0.19 (0.12, 0.26)
Load	0.04 (0.02, 0.07)	0.10 (0.05, 0.16)	0.07 (0.03, 0.12)	0.19 (0.12, 0.25)

##### Proportion of time

As with the proportion of fixations, for the proportion of time, no main effect of Location, *F*(1, 45) = 1.18, *p* = .28, ${\eta}_G^2$ <.01, BF_inc_ = 0.34, and no significant Condition by Location interaction, *F*(1, 45) = 2.14, *p* = .15, ${\eta}_G^2$ <.01, BF_inc_ = 1.23, were observed. A significant interaction between Proximity and Location, *F*(1, 45) = 9.32, *p* = .004, ${\eta}_G^2$ =.02, BF_inc_ = 4.01, was observed, such that when fixating toward the confederate’s head, no significant difference in the proportion of time spent fixating across the near (*M* = 0.13) and far (*M* = 0.10) conditions, *t*(45) = 0.87, *p =* .39, *d* = 0.15, BF_10_ = 0.24, was observed, but when looking at the confederate’s body, the participants spent less time spent fixating when near (*M =* 0.10), compared to when far (*M =* 0.19), *t*(45) = 3.26, *p =* .002, *d* = 0.56, BF_10_ = 6.67. No significant three-way interaction, *F*(1, 45) = 3.12, *p* = .08, ${\eta}_G^2$ <.01, BF_inc_ = 0.72, was observed.

### Discussion

Experiment 2 largely replicated the main results of Experiment 1. Specifically, in the load condition compared to the no-load condition, participants look less at the confederate. Unlike in Experiment 1, no main effect of proximity was observed; however, a significant interaction was observed between load and proximity, such that the effect of cognitive load was present only when the participant was near the confederate. Indeed, in the no load condition, unlike Experiment 1, no overall reduction in looks toward the confederate was observed, but a clear reduction in the load condition was observed. As in Experiment 1, a significant proximity by location interaction was also observed, such that, overall, looks toward the body decreased with increasing proximity, but looks toward the head increased (though the Bayes was anecdotal for the analysis of looks toward the head). The Bayes factors associated with these results were mainly consistent with the conclusions drawn. Overall, the influence of cognitive load on looks toward a pedestrian seems reliable. In Experiment 3, we look to further examine the mechanism underlying this effect.

## Experiment 3

In both Experiments 1 and 2, clear evidence existed that adding a cognitive load reduced looks toward another social agent. Why might the role of cognitive load take this specific form? In the Introduction, we arrived at the idea that cognitive load might influence social looking by considering the dual functions of looking behavior. Individuals need to acquire information from other social agents by looking at them, but at the same time, they also need to monitor social norms associated with their gaze (i.e., need to monitor what they communicate with their gaze). The need to manage these two functions seemingly arises only in authentic social contexts and arguably puts pressure on a limited capacity executive control system. This argument makes a straightforward prediction. Specifically, the influence of cognitive load should be reduced/eliminated in a less authentic social context where the need to manage the two functions is less pressing. We tested this prediction in Experiment 3 by examining the influence of cognitive load on social attentional behavior using prerecorded social stimuli. Specifically, we replicated Experiment 2, but instead of having individuals walk a predetermined route and encounter a real social agent, we recorded a video of someone taking that walk and presented it to the participant as a video (displayed on a large screen to keep the approximate dimensions the same). Thus, we created a similar visual experience, but the confederate the participants encountered was not a live social agent. Similar methods have been used in previous work to manipulate the dual function of gaze (see Foulsham et al., 2011; Laidlaw, Foulsham, Kuhn, & Kingstone, 2011).

If the load effects observed in Experiment 1 and 2 reflect the needs to manage the putative dual functions of gaze, then there should be no effect of cognitive load in Experiment 3. In addition, as mentioned in the introduction, Foulsham et al. (2011) demonstrated that changes in gaze as a function of proximity were modulated by whether individuals were embedded in the social context (i.e., actually walking on campus) versus not. Experiment 3 provides (a) an opportunity to replicate that general effect (through comparison to Experiment 2) and (b) an opportunity to explore the extent to which the underlying mechanism is such that load either reduces or exacerbates it.

### Methods

#### Participants

A total of 62 undergraduate students from the University of Waterloo completed this study for course credit. Thirteen participants were removed, one participant was excluded for health reasons, and the other 12 for technical issues (e.g., issues with the video playing, calibration issues, or issues with recording), resulting in 49 participants remaining. The same power analysis as Experiment 2 was used for this experiment.

#### Stimuli

Similar to Experiment 2, participants completed both the no-load and load conditions. However, in this experiment, participants were told to stand (but not walk) in an empty room, where they watched the projected video of two different walking routes. The projector screen was 2.1 m wide and 1.6 m tall. Participants stood anywhere from 1.8 to 2.4 m away from the screen; this varied due to the participant height as a way to maintain a similar visual angle for all participants. The video was created of the same two walking routes the participants in Experiment 2 experienced. The video was shot in first person, from the vantage point of a participant, to simulate what participants would have seen in Experiment 2. For a fluid video without obvious head movements, the video was filmed by rolling the camera down the hall rather than holding it. Each of the two walking route videos was approximately 2 min long filmed with 1080i at 60 frames per second. Given the two different routes, two confederates were filmed. The total walking time for one of the hallways was 42 s, with the confederate in view for 17 s. For the other hallway walk, the total time of the walk was 36 s, with the confederate in view for 15 s. Both confederates were female and walked down each of the two routes. The two confederates in this experiment were filmed looking down and away from the camera, since looking into the camera would have simulated eye contact. The background lighting in the room was turned off to ensure nothing besides the screen would be seen. The N-back task used was the same as in Experiments 1 and 2.

After participants watched both videos, they were asked to complete a Social Phobia Inventory Scale (Connor et al., 2000). This scale had 17 questions asking participants to rate how bothered they were by social situations within the last week according to a scale of 0 (Not at all) to 4 (Extremely). For example, one statement is “I avoid going to parties”, which the participant can answer from not at all to extremely. This scale has been shown to have a high (> 85%) specificity and sensitivity for assessing generalized social anxiety disorder (Connor et al. 2000). These data are not reported here since they were inaccessible due to the COVID-19 pandemic at the time of publication.

#### Apparatus

The apparatus was the same as in Experiments 1 and 2, with the addition of an Epson high definition projector, which was used to project the two videos of the walking routes. In addition, a large projection screen (2.1 × 1.6 m) was used so that the objects in the video displayed at a size similar to what they would have, had the participant actually experienced the recorded scene.

#### Procedure

The procedure was similar to the first two experiments. Participants were fitted with the mobile eye tracker and headphones; however, rather than walk, the participants stood in the center of the room facing the screen. The eye tracker was calibrated as in the previous experiments. Once the tracker was calibrated, the participants watched the video of the two walking routes while completing the no-load and load conditions. Which task the participant completed for the first route was counterbalanced across participants. Once a participant had watched both walking routes, the eye tracker was removed, and the participant completed the social phobia scale and was debriefed.

### Results

Data were coded in the same manner as in Experiments 1 and 2.

#### Non-eye movement measures

##### N-back accuracy

N-back data were missing for two of the participants since they never received a target letter (see Table 1).

#### Eye-movement measures – on vs off the confederate

The proportion of fixations, proportion of time, number of fixations, and total time were recorded for fixation behavior toward the confederate (see Table 6). Importantly, no effect of order or of which hallway walk was shown first, was observed for any of the fixation variables (all *p*’s > .05).

**Table 6:** Fixation behavior toward the confederate grouped by condition (no-load, load) and Proximity (near, far) in Experiment 3, time is reported in seconds

	Near	Far
	Mean (95%CI)	Mean (95%CI)
***Proportion of Fixations***		
No-load	0.35 (0.26, 0.45)	0.17 (0.11, 0.23)
Load	0.24 (0.17, 0.31)	0.20 (0.14, 0.26)
***Proportion of Time***		
No-load	0.39 (0.28, 0.49)	0.23 (0.15, 0.31)
Load	0.23 (0.16, 0.30)	0.25 (0.17, 0.33)
***Number of Fixations on the Confederate***		
No-load	1.59 (1.13, 2.06)	2.22 (1.50, 2.95)
Load	1.14 (0.80, 1.48)	2.43 (1.67, 3.19)
***Time Fixating on the Confederate***		
No-load	0.95 (0.67, 1.24)	1.95 (1.23, 2.66)
Load	0.52 (0.36, 0.68)	1.73 (1.23, 2.24)
***Total Fixations***		
No-load	4.35 (3.65, 5.04)	14.90 (12.68, 17.11)
Load	4.86 (4.28, 5.43)	13.88 (12.11, 15.65)
***Total Time***		
No-load	2.29 (2.06, 2.53)	7.14 (6.32, 7.95)
Load	2.25 (2.10, 2.40)	7.00 (6.25, 7.74)

##### Proportion of fixations

**Fig. 4:**
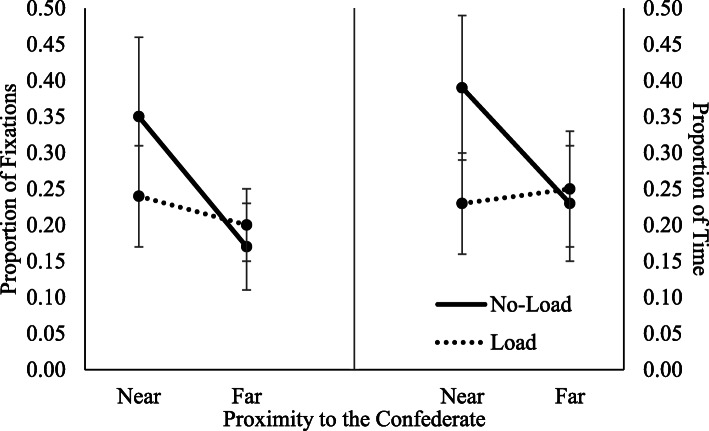
The mean proportion of fixations (left panel) and time spent on the confederate (right panel) when the confederate was in view for Condition (no-load, load) and Proximity (near, far) in Experiment 3. Bars show 95% confidence intervals

We conducted a Condition by Proximity repeated measures ANOVA on the proportion of fixations directed toward the confederate. No significant main effect of Condition, *F*(1,48) = 1.62, *p* = .21, ${\eta}_G^2$ <.01, BF_inc_ = 0.34, was observed. A significant main effect of Proximity, *F*(1,48) = 12.24, *p* = .001, ${\eta}_G^2$ =.05, BF_inc_ = 41.40, was observed, such that there was a higher proportion of fixations on the confederate when near (*M* = 0.30) compared to when far (*M =* 0.18). Additionally, a significant interaction was observed between Condition and Proximity, *F*(1,48) = 5.96, *p* = .02, ${\eta}_G^2$ =.02, BF_inc_ = 1.88, such that when near, individuals had a higher proportion of fixations toward the confederate in the no-load condition (*M* = 0.35) compared to the load condition (*M* = 0.24), *t*(48) = 2.19, *p* = .03, *d* = 0.40, BF_10_ = 1.37; however, when far, no difference was observed between the no-load (*M* = 0.17) and load conditions (*M* = 0.20), *t*(48) = 0.71, *p* = .48, *d* = 0.13, BF_10_ = 0.20 (see Fig. 4). Importantly, the condition by proximity interaction, while significant, was associated with a Bayes factor that revealed only “anecdotal” evidence.

##### Proportion of time

Similar to the proportion of fixations, for the proportion of time fixating on the confederate, the main effect of Condition was not significant, *F*(1,48) = 3.68, *p* = .06, ${\eta}_G^2$ =.01, BF_inc_ = 0.82. Unlike proportion of fixations, no significant main effect of Proximity, *F*(1,48) = 3.13, *p* = .08, ${\eta}_G^2$ =.01, BF_inc_ = 0.69, was observed. Again, a significant interaction between Condition and Proximity, *F*(1,48) = 6.04, *p* = .02, ${\eta}_G^2$ =.02, BF_inc_ = 2.78, was observed, such that when near, participants fixated on the confederate for a higher proportion of time in the no-load condition (*M =* 0.39) compared to the load condition (*M* = 0.23), *t*(48) = 2.68, *p* = .01, *d* = 0.50, BF_10_ = 3.75; however, when far, no difference was observed between the no-load (*M =* 0.23) and load (*M =* 0.25) conditions, *t*(48) = 0.39, *p* = .70, *d* = 0.06, BF_10_ = 0.17 (see Fig. 4). As with the proportion of fixations, the condition by proximity interaction, while significant, was associated with a Bayes factor that revealed only “anecdotal” evidence.

#### Eye-movement measures- head vs. body

##### Proportion of fixations

We conducted a Condition by Proximity by Location repeated measures ANOVA on the proportion of fixations toward the confederate. No significant main effect of Location, *F*(1,48) = 0.80, *p* = .38, ${\eta}_G^2$ <.01, BF_inc_ = 0.20, was observed. A significant interaction between Condition and Location, *F*(1,48) = 4.69, *p* = .04, ${\eta}_G^2$ =.01, BF_inc_ = 2.22, was observed, such that a significantly higher proportion of fixations toward the confederate’s head were observed in the no-load condition (*M =* 0.16) compared to the load condition (*M* = 0.10), *t(*48) = 2.45, *p* = .02, *d* = 0.43, BF_10_ = 2.30, but no significant difference was observed when looking at proportion of fixations toward the confederate’s body (no-load *M* = 0.10, load *M =* 0.12), *t*(48) = 0.81, *p* = .42, *d* = 0.15, BF_10_ = .021. A significant interaction was also observed between Proximity and Location, *F*(1,48) = 15.95, *p* < .001, ${\eta}_G^2$ =.04, BF_inc_ = 423.67, such that the proportion of looks toward the head were greater when near (*M =* 0.20) compared to when far (*M =* 0.06), *t*(48) = 5.77, *p* < .001, *d* = 0.92, BF_10_ = 28,869.44; however, no significant difference was observed for the proportion of fixations toward the confederate’s body when near (*M =* 0.10) compared to when far (*M =* 0.12), *t*(48) = 0.75, *p* = .46, *d* = 0.14, BF_10_ = 0.20. No three-way interaction, *F*(1,48) = 3.86, *p* = .06, ${\eta}_G^2$ <.01, BF_inc_ = 1.08, (see Table 7) was observed.

**Table 7:** Proportion of fixations toward the head and body for Condition (no-load, load) and Proximity (near, far) in Experiment 3

	Head		Body	
	Near	Far	Near	Far
	Mean (95%CI)	Mean (95%CI)	Mean (95%CI)	Mean (95%CI)
***Proportion of fixations***				
No-load	0.27 (0.18, 0.35)	0.06 (0.03, 0.09)	0.09 (0.03, 0.15)	0.11 (0.06, 0.16)
Load	0.13 (0.08, 0.18)	0.07 (0.03, 0.10)	0.11 (0.05, 0.17)	0.13 (0.08, 0.18)
***Proportion of time***				
No-load	0.28 (0.18, 0.38)	0.08 (0.04, 0.12)	0.10 (0.04, 0.17)	0.15 (0.08, 0.22)
Load	0.12 (0.06, 0.18)	0.08 (0.03, 0.13)	0.11 (0.05, 0.16)	0.17 (0.10, 0.23)

##### Proportion of time

When we examined the proportion of time spent fixating toward the confederate, we found no significant main effect of Location, *F*(1,48) = .010, *p* = .75, ${\eta}_G^2$ <.01, BF_inc_ = 0.12. In addition, no Condition by Location interaction, *F*(1,48) = 3.58, *p* = .07, ${\eta}_G^2$ =.01, BF_inc_ = 1.01, was observed. A significant Proximity by Location interaction, *F*(1,48) = 12.98, *p* < .001, ${\eta}_G^2$ =.04, BF_inc_ = 252.19, was observed, such that when near (*M =* 0.20), participants spent proportionally more time fixating on the confederate’s head, compared to when far (*M* = 0.08), *t*(48) = 4.56, *p* < .001, *d* = 0.69, BF_10_ = 604.38. In contrast, no difference was observed in the proportion of time spent fixating on the confederate’s body when near (*M =* 0.11) versus far (*M* = 0.16), *t*(48) = 1.57, *p* = .12, *d* = 0.31, BF_10_ = 0.49. No significant three-way interaction, *F*(1,48) = 3.41, *p* = .07, ${\eta}_G^2$ <.01, BF_inc_ = 0.78 (see Table 7) was observed.

### Discussion

Overall, with respect to the influence of cognitive load, we found a similar, though less robust (when considering the Bayesian analysis), pattern of results in Experiment 3, as we did in Experiments 1 and 2. Specifically, an effect of load was observed, such that participants fixated less on the confederate in the load condition, compared to the no-load condition, only when proximally near the confederate. With respect to load, these results are inconsistent with the hypothesis that the load effect in Experiments 1 and 2 was a byproduct of the need, in an authentic social context, to manage both the desire to acquire information and the need to manage what one’s gaze communicates to others. In particular, the latter was not a consideration in Experiment 3, yet a similar effect of load emerged. We return to alternative explanations of this effect in the General Discussion.

While the overall pattern of results with respect to cognitive load were similar across Experiments 1, 2 and 3, one salient difference existed across the more (Experiment 1 and 2) and less authentic (Experiment 3) situations. Namely, in Experiment 3, the effect of proximity reflected an increase in overall looks at the confederate as they drew closer. No significant overall effect of proximity was observed in Experiment 2, and in Experiment 1, looks toward the confederate declined when near. Foulsham et al. (2011) reported a qualitatively similar pattern in that the difference between a more and less authentic context was most pronounced in the near condition. In Foulsham et al. (2011), this reflected a less pronounced reduction in looks toward the other pedestrian in the video condition, whereas here, an increase was observed in the video condition, but no increase (E2) or a decrease (E1) was observed in the “live” condition. Importantly, in both cases, a relative reduction in looks to people when in close proximity was observed in authentic social contexts. We examine this putative interaction further in a combined analysis. Lastly, as in Experiments 1 and 2, a significant proximity by location interaction was observed. The increase in looks toward the confederate in the near condition overall was restricted to looks toward the head, and a non-significant reduction in looks toward the body remained as individuals drew near. As in Experiments 1 and 2, the Bayes factors associated with these results were consistent with the conclusions drawn. That said, the interaction between condition and proximity in Experiment 3, while significant, was associated with only anecdotal evidence based on the Bayes factors reported.

### Combined analysis of experiments 2 and 3

The similarity between Experiments 2 and 3 provides an opportunity to evaluate a number of issues. As such, we conducted an Experiment (2, 3) by Condition (no-load, load) by Proximity (near, far) by Location (head, body) mixed ANOVA on both the proportion of fixations and the proportion of time spent looking at the confederate. Importantly, causal inferences are not appropriate in the following analysis since participants are not randomly assigned to conditions.

#### Proportion of fixations

The proportion of fixations toward the confederate were analyzed with an Experiment by Condition by Proximity by Location mixed ANOVA. No significant main effect of Experiment, *F*(1, 93) = 0.01, *p* = .90, ${\eta}_G^2$ <.01, BF_inc_ = 0.11, and no significant Condition by Experiment interaction, *F*(1, 93) = 0.85, *p* = .36, ${\eta}_G^2$ <.01, BF_inc_ = 0.17, were observed. In addition, no significant interactions between Condition, Proximity, and Experiment, *F*(1, 93) = 0.34, *p* = .56, ${\eta}_G^2$ <.01, BF_inc_ = 0.15, or between Condition, Location and Experiment, *F*(1, 93) = 0.32, *p* = .57, ${\eta}_G^2$ <.01, BF_inc_ = 0.17, were observed . In addition, no four-way interaction was observed between Condition, Proximity, Location and Experiment, *F*(1, 93) = 0.14, *p* = .71, ${\eta}_G^2$ < .01, BF_inc_ = 0.14. The latter interactions confirm that the effect of load on looking did not change across the more and less authentic contexts.

**Fig. 5:**
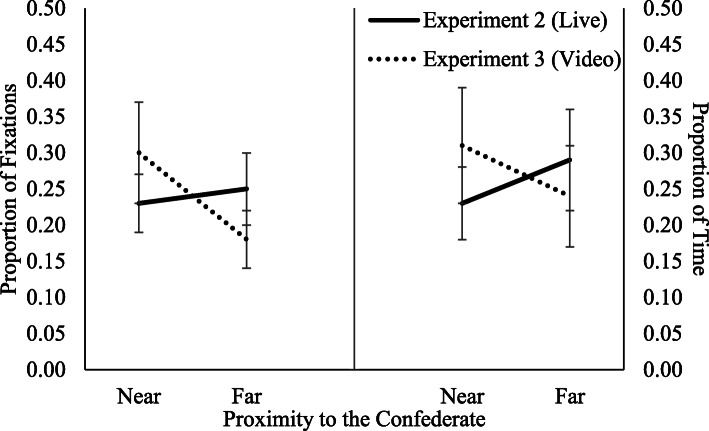
The mean proportion of fixations (left panel) and time spent on the confederate (right panel) when the confederate was in view for Proximity (near, far) as a function of Experiment (2 and 3). Bars represent 95% confidence intervals

While no effect was observed of Experiment on the influence of condition, a significant interaction was observed between Proximity and Experiment, *F*(1, 93) = 8.17, *p* = .005, ${\eta}_G^2$ < .01, BF_inc_ = 3.92 (see Fig. 5), such that those in Experiment 2 showed no significant effect of Proximity to the confederate (near *M* = 0.23; far *M =* 0.25)*, t*(45) = 0.62, *p* = .54, *d* = 0.12, BF_10_ = 0.19; however, in Experiment 3, when near (*M* = 0.30*),* the proportion of fixations toward the confederate were significantly higher than when far (*M =* 0.18), *t*(48) = 3.50, *p* = .001, *d* = 0.58, BF_10_ = 28.21. No significant interaction was observed between Location and Experiment, *F*(1, 93) = 2.63, *p* = .11, ${\eta}_G^2$ < .01, BF_inc_ = 1.03 and no significant interaction was observed between Proximity, Location and Experiment, *F*(1, 93) = 0.66, *p* = .42, ${\eta}_g^2$ < .01, BF_inc_ = 0.19.

Lastly, a significant Location by Condition interaction was observed for the proportion of fixations on the confederate, *F*(1, 93) = 7.77, *p* = .006, ${\eta}_G^2$ = .01, BF_inc_ = 6.14, such that the effect of load was only significant for fixations toward the confederate’s head, *t*(94) = 4.32*, p* < .01, *d* = 0.53, BF_10_ = 462.76, but not the body, *t*(94) = 0.20, *p* = .84, *d* = .03, BF_10_ = 0.12.

#### Proportion of time

When we examined the proportion of time, no significant main effect of Experiment, *F*(1, 93) = 0.15, *p* = .69, ${\eta}_G^2$ < .01, BF_inc_ = 0.11, and no interaction between Experiment and Condition, *F*(1, 93) = 0.64, *p* = .43, ${\eta}_G^2$ < .01, BF_inc_ = 0.13, were observed. In addition, no interaction was observed between Condition, Proximity, and Experiment, *F*(1, 93) = 0.20, *p* = .66, ${\eta}_G^2$ < .01, BF_inc_ = 0.16, and no interaction was observed between Condition, Location, and Experiment, *F*(1, 93) = 0.30, *p* = .59, ${\eta}_G^2$ < .01, BF_inc_ = 0.14. In addition, no four-way interaction between Condition, Proximity, Location, and Experiment, *F*(1, 93) = 0.10, *p* = .75, ${\eta}_G^2$ < .01, BF_inc_ = 0.28, was observed.

Again, a significant Proximity by Experiment interaction, *F*(1, 93) = 6.05, *p* = .02, ${\eta}_G^2$ < .01, BF_inc_ = 0.94, was observed. While no effect of proximity was observed in Experiment 2, *t*(45) = 1.71, *p* = .09, *d* = 0.31, BF_10_ = 0.53, or in Experiment 3, *t*(48) = 1.77, *p* = .08, *d* = 0.29, BF_10_ = 0.66, the interaction reflects the opposing direction of the trends in each experiment. No significant interaction was observed between Location and Experiment, *F*(1, 93) = 0.94, *p* = .34, ${\eta}_G^2$ < .01, BF_inc_ = 0.29, and no significant interaction was observed between Proximity, Location, and Experiment, *F*(1, 93) =1.25, *p* = .27, ${\eta}_G^2$ < .01, BF_inc_ = 0.34.

Similar to the proportion of fixations, a significant Location by Condition interaction for proportion of time spent looking at the confederate, *F*(1, 93) = 5.63, *p* = .02, ${\eta}_G^2$ < .01, BF_inc_ = 6.64, was observed, such that there was a significant difference between the no-load and load conditions when looking at the confederate’s head, (no-load *M* = 0.17, load *M* = 0.09), *t*(94) = 4.35, *p* < .001, *d* = 0.51, BF_10_ = 452.48 but not when looking toward the body (no-load *M* = 0.14, load *M* = 0.13), *t*(94) = 0.39, *p* = .69, *d* = 0.05, BF_10_ = 0.12.

**Fig. 6:**
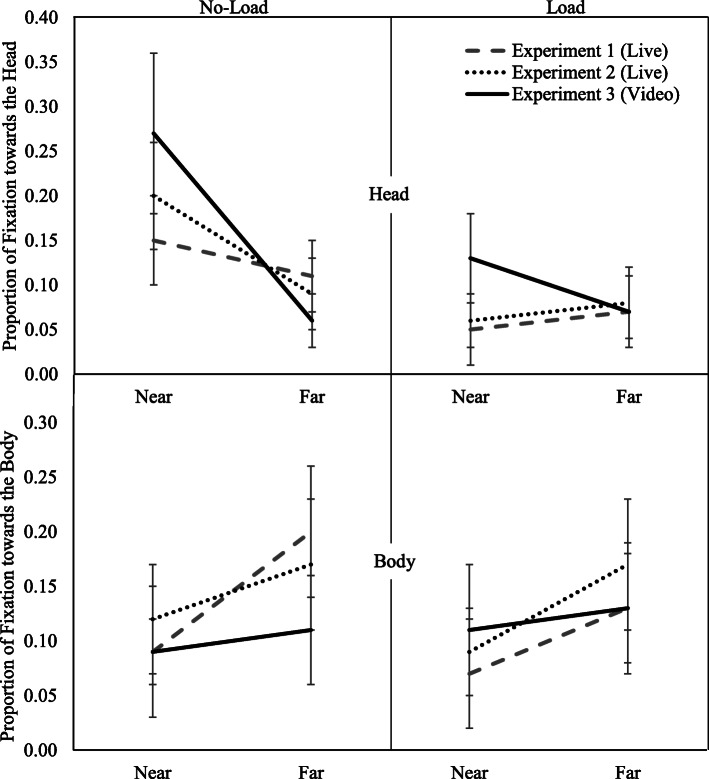
The mean proportion of fixations as a function load, location and Experiment. Bars represent 95% confidence intervals

The results of the foregoing combined analysis demonstrated that, across Experiments 2 and 3, the influence of cognitive load on looks toward the confederate was similar and that this effect was most pronounced for looks toward the head. There was, however, a difference in the influence of proximity on fixation behavior. In the more authentic condition, with a real pedestrian passing, no overall effect of proximity was observed on the participant’s gaze toward the social agent as the agent approached. When that social agent was simply an image of a social agent, the individuals increased their gaze toward the confederate as the confederate approached. Critically, this interaction was not modulated by load. That is, the interaction took qualitatively the same form, whether the individuals were walking under load or not. This suggests that the cause of this particular phenomenon (i.e., the change in the proximity effect as a function of “live” vs. “video” stimuli) is not related to resource availability. To provide a clear depiction of the results across Experiments, Figs. 6 (for proportion of fixation) and 7 (for proportion of time) plot each dependent variable as a function of Experiment, load, and location of looks.

**Fig. 7:**
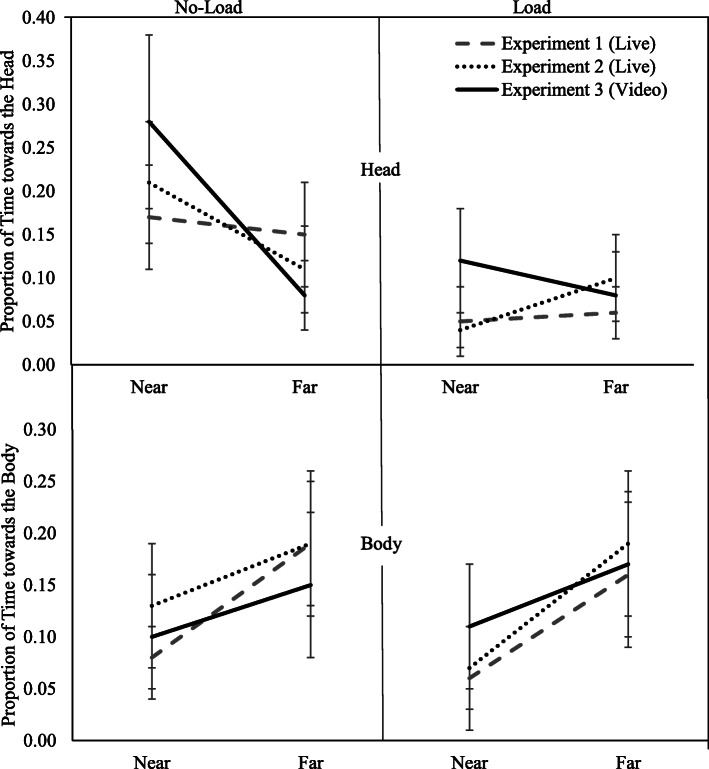
The mean time spent on the confederate when the confederate was in view as a function of load (no load, load), location (head, body), and Experiment (Experiment 1, 2, 3). Bars represent 95% confidence intervals

Importantly, the interaction between proximity and experiment provides evidence that the “live” vs. “video” manipulation of the authenticity of the situation was effective (i.e., it clearly influenced gaze behavior) across Experiments 2 and 3. This makes the lack of a change in the influence of cognitive load across these experiments particularly troublesome for the hypothesis, which we proposed, that the load effect was in some manner a by-product of the authenticity of the social contexts in Experiments 1 and 2.

## General discussion

Based on a prediction derived from the dual function of gaze framework, we examined, in three mobile eye tracking experiments, the influence of cognitive load on social attentional behavior across more and less authentic social contexts. In each experiment, we found evidence consistent with the idea that cognitive load influences social attentional behavior. Specifically, under a higher cognitive load, looks toward another social agent decreased. This was replicated three times, was true in both a between- and within-subject design, and was true when the pedestrian actually performed the walk or watched a video equivalent. This effect appears strongest when the two social actors were near one another (the interaction was present in Experiments 2 and 3 but not Experiment 1), and this effect seems to be restricted to a reduction in looks toward the head.

### The influence of cognitive load on social looking

The motivation for the present investigation into load effects on social attentional behavior was derived from the need, in an authentic social context, to weigh both the informational value of fixating a given location and the social consequences of doing so. While we confirmed, across three separate experiments, that there is indeed a robust effect of cognitive load on social looking, attributing it to a need to manage these dual functions seems inconsistent with the results of Experiment 3. Namely, less need should exist for such management when the social agent is not real (and thus cannot enact any social consequences for our fixation behavior).

What can account for the observed effect of cognitive load? In addition to revealing a significant effect of cognitive load on social looking, our experiments also revealed a number of patterns (some more robust than others) that could help constrain the search for the underlying mechanism. Namely, the effect of cognitive load is a reduction (not an increase) in looks toward a social agent, the effect seems more robust near a social agent and seems to be limited to looks toward the head, and the effect is present in both a socially authentic context and an inauthentic one (i.e., watching a video). One potential explanation for the effect of cognitive load on social looking is that it reflects a kind of cognitive control strategy, specifically a form of gaze aversion (Abeles & Yuval-Greenburg, 2017; Doherty-Sneddon et al., 2002; Glenberg et al., 1998). As mentioned previously, Doherty-Sneddon et al. (2002) demonstrated that children reduce looks toward an experimenter in a face to face interaction while completing challenging cognitive tasks. The authors argue that the higher load caused by the more challenging questions drove the participants to avert their gaze to minimize cognitive load and allow the children to concentrate their resources on the challenging task. This disengagement is viewed as a means of avoiding cognitive overload (Doherty-Sneddon, 2004; Doherty-Sneddon et al., 2002; Doherty-Sneddon & Phelps, 2005). Viewed from this perspective, the effect of cognitive load here could be viewed as a form of gaze aversion. This would explain the reduction in looks toward the social agent under load. That is, under load, the participants avoid looking at the confederate to avoid the potential overload that might occur if the social agent is processed. In addition, the restriction of this effect to near space and to the head seem explicable. In near space, the confederate’s face would be resolvable, and likewise, the head is the high information location within the social agent. Lastly, if we consider a face (live or videotaped) to be highly salient and informative, then the lack of a difference between authentic and inauthentic scenarios also makes theoretical sense.

An alternative idea is that the load-based avoidance of the social agent had little to do with the social status of that agent and had more to do with the social agent’s mere physical presence in the space. According to this idea, we might have found similar results if, instead, we had placed an object in the hallway (e.g., a chair). This is an interesting idea to consider, and the present design does not allow for a clear rejection of it. That said, the pattern across Experiments 1, 2, and 3 (see Tables 3, 5 and 7 and the combined analysis) suggests that the reduction in looks as a function of load was restricted largely to looks toward the head rather than looks toward the body. That is, there did not appear to be much impact of cognitive load on looks toward the body. This seems inconsistent with the idea that load reduces looks toward any object in physical space. Future research including such nonsocial objects would be valuable.

### Proximity

Beyond the effect of cognitive load, the experiments also demonstrated changes in looking behavior as a function of proximity. First, in Experiments 1 and 2, gaze toward the confederate remained constant (or decreased) as participants drew nearer to the confederate. In Experiment 3, gaze toward the confederate increased under the same conditions. When divided into looks between the head and body of the confederate, a clear pattern emerged (e.g., see Tables 3, 5 and 7; Figs. 6 and 7) such that the closer one came to the confederate, the less one looked toward the body and the more one looked toward the head. Critically, as noted above, cognitive load interacted with these proximity effects and load appeared to selectively influence looks toward the head; leading to a pronounced reduction in the proportion of looks toward the confederate when near in Experiments 1 and 2, and a muting of the increase in the proportion of looks toward the confederate when near in Experiment 3. Taken together, this finding suggests that changes in gaze patterns as a function of proximity are at least partially related to resource demands as articulated in the gaze aversion account of the load effects above. In addition, there appears to be a general decline in gazes toward the body when individuals are close, and this decline does not appear to be (strongly) modulated by load. This might reflect a decreasing need to monitor the individual’s trajectory in order to avoid collision. That is, once someone has been tracking an individual for a period of time, their path would be clear. It is also important to note that any increase in looks toward the head could draw looks, proportionally, away from the body. Lastly, provided the position of the participant’s head relative to the pedestrian’s body, as the two individuals approach one another, the relative size of each other’s head, as projected on the retina, would likely increase relative to the body. The latter might also influence the distribution of gaze.

### Dual function of gaze

The present investigation took the dual function perspective as a starting point for understanding social attentional behavior and motivating the investigation of potential cognitive load effects. As noted above, given the results of Experiment 3, the load effect observed here does not appear to result from the management of the dual functions of gaze. This might suggest that the management of those dual functions do not put demands on the cognitive control system (or at least demands on the cognitive control system that are disrupted by an N-back task). This conclusion, however, may be premature. As noted in the Introduction, cognitive control is typically required when there is competition in information processing (e.g., inhibiting a pre-potent response). The pedestrian passing scenario created here may not have put the information acquisition and communicative functions in competition. In addition, while the social context used here is more naturalistic than looking at static social scenes, room exists to increase the amount of social interaction required (e.g., a conversation). Future research creating such a situation may well reveal cognitive load effects that can be explicitly tied to managing the dual functions of gaze. Indeed, while Experiments 1–3 reveal seemingly consistent patterns across the cognitive load manipulation, this is the first such investigation and replication (e.g., pre-registered, independent) would be valuable. This will be particularly important for the putative moderators of the load effects (e.g., by distance, head vs. body) or lack thereof (e.g., live vs. video).

## Conclusion

Across these three experiments, we demonstrate that cognitive load modulates social attentional behavior. This was demonstrated in both an authentic and inauthentic social context and provides a novel look into potential interactions between cognitive control and social attention. Future research examining the mechanism proposed here (i.e., gaze aversion as a form of cognitive control) promises deeper insight into how individuals manage their capacity limitations in social situations and beyond.

## Supplementary information

**Additional file 1.**

## Data Availability

The datasets and analyses are avaiable on the Open Science Framework (OSF) here: https://osf.io/c2hd8/.
