# Supplementary material for: The role of cognitive load in modulating social looking: a mobile eye tracking study
Source: Cogn Res Princ Implic. 2020 Sep 16;5:44. doi: 10.1186/s41235-020-00242-5 (PMC7493067; doi:10.1186/s41235-020-00242-5)
Supplement: Supplementary file 1 — Additional file 1. [file 41235_2020_242_MOESM1_ESM.docx]

**Supplemental Materials:**

**Part 1:** Analysis of the Social Desirability scale administered in Experiments 1 and 2.

Participants completed a social desirability scale after completing the walking task. This questionnaire was scored out of 33 with higher scores representing individual’s reporting more socially desirable actions (Crowne & Marlowe, 1960). While we provide these correlations in both Experiment 1 and 2, it is important to note that this measure was included for exploratory purposes, and sample size is limited with regards to interpreting correlations or lack thereof. As such, these results are restricted in what they can tell us about social desirability and looking behaviour.

*Experiment 1.*

There was no difference in social desirability ratings for those assigned to the load and no load conditions, t(78)=0.43, *p*=0.67, d=0.09. The mean for the load condition was 15.45 (*SD*=5.36) and the mean for the no load condition was 15.92 (*SD*=4.18). We also examined correlations between scores on the social desirability scale and both the proportion of fixations and the proportion of time spent fixating on the confederate.

*Proportion of Fixations.* Social desirability did not significantly correlate with the proportion of fixations on the confederate overall, *r*(78)=-0.12, *p*=.31. The same was true when we examined those in the load condition, *r*(36)=0.01, p=.96, and those in the no load condition, *r*(38)=-.2, *p*=.20, separately. Similarly, social desirability scores did not correlate with the proportion of fixations on the confederate when near, *r*(78)=-.14, *p*=.23, or far, *r*(78)=-.04, *p*=.71.

*Proportion of Time.* Social desirability did not significantly correlate with the proportion of time spent fixating on the confederate overall, *r*(78)=-.07, *p*=.51. The same was true when we examined those in the load condition, *r*(36)=-.01, p=.94, and those in the no load condition, *r*(38)=-.10, *p*=.53, separately. Again, social desirability scores did not correlate with the proportion of time spent fixating on the confederate when near, r(78)=-.11, *p*=.31, or far, r(78)=-.007, *p*=.95.

*Experiment 2.*

The mean social desirability for participants in Experiment 2 was 15.81 (*SD*=3.78). We again examined correlations between scores on the social desirability scale and both the proportion of fixations and the proportion of time spent fixating on the confederate.

*Proportion of Fixations.* Social desirability scores did not significantly correlate with the proportion of fixations on the confederate overall, *r*(45)=.09, *p*=.53. The same was true when we examined the proportion of fixations in the load condition, *r*(45)=.18, p=.22, and in the no load condition, *r*(45)=-.02, *p*=.88, separately. Similarly, regardless of load condition, social desirability scores did not correlate with the proportion of fixations on the confederate when near, *r*(45)=.13, *p*=.39, or far, *r*(45)=.02, *p*=.90.

*Proportion of Time.* Social desirability did not significantly correlate with the proportion of time spent fixating on the confederate overall, *r*(45)=.08, *p*=.62. The same was true when we examined the proportion of time fixating during the load condition, *r*(45)=.23, p=.12, or during the no load condition, *r*(45)=-.09, *p*=.53, separately. Similarly, regardless of load condition, social desirability scores did not correlate with the proportion of fixations on the confederate when near, *r*(45)=.09, *p*=.53, or far, *r*(45)=.03, *p*=.83.

**Part 2:** Analysis of the additional manipulation in Experiment 1.

In Experiment 1 there was another confederate that was seated in a chair either facing towards or away from the participant on a separate part of the route the participant travelled, some saw an empty chair. This manipulation was not intended to influence behaviour on the hallway examined here but we provide an analysis including this factor out of an abundance of caution. We conducted a Condition (load, no load) by Proximity (near, far) by Chair Direction (facing forward, facing away, empty chair) mixed ANOVA for both for the proportion of fixations and proportion of time spent fixating on the walking confederate.

*Proportion of Fixations:* There was no significant main effect of Chair Direction *F*(2, 74)=0.07, *p*=.93, η^2^_g_=.00, BF_inc_=0.09, and no interaction between Chair Direction and Condition, *F*(2, 74)=2.11, *p*=.13, η^2^_g_=.02, BF_inc_=0.62, or Proximity and Chair Direction, *F*(2, 74)=0.22, *p*=.81, η^2^_g_=.00, BF_inc_=0.13. There was also no 3-way interaction between Condition, Proximity and Chair Direction, *F*(2, 74)=0.33, *p*=.72, η^2^_g_=.00, BF_inc_=0.23, see Table S1.

*Proportion of Time:* There was no significant main effect of Chair Direction *F*(2, 74)=0.07, *p*=.93, η^2^_g_=.00, BF_inc_=0.10, and no interaction between Chair Direction and Condition, *F*(2, 74)=2.32, *p*=.11, η^2^_g_=.02, BF_inc_=0.68, or Proximity, *F*(2, 74)=0.21, *p*=.81, η^2^_g_=.00, BF_inc_=0.12. There was also no 3-way Condition by Proximity by Chair Direction interaction, *F*(2, 74)=0.47, *p*=.62, η^2^_g_=.00, BF_inc_=0.23, see Table S1.

Table S1: *Means and 95% confidence intervals as a function of Chair Direction, for both proportion of fixations and proportion of time, across Condition, and Proximity.*

|  | **Load** | | | | |  | **No Load** | | |  |
| --- | --- | --- | --- | --- | --- | --- | --- | --- | --- | --- |
|  | **Near** | | |  | **Far** |  | **Near** |  | **Far** |  |
|  | Mean [95%CI] | | |  | Mean [95%CI] |  | Mean [95%CI] |  | Mean [95%CI] |  |
| ***Proportion of Fixations*** | | |  |  |  |  |  |  |  |  |
| Chair Forward | 0.07 [-0.00,0.14] | | |  | 0.16 [0.06,0.27] |  | 0.26 [0.20,0.33] |  | 0.35 [0.21,0.48] |  |
| Chair Away | 0.09 [0.01,0.16] | | |  | 0.21 [0.08,0.34] |  | 0.25 [0.12,0.39] |  | 0.29 [0.19,0.40] |  |
| Empty Chair | 0.20 [0.01,0.39] | | |  | 0.22 [0.07,0.37] |  | 0.21 [0.10,0.31] |  | 0.27 [0.15,0.39] |  |
| ***Proportion of Time*** | |  | |  |  |  |  |  |  |  |
| Chair Forward | 0.06 [-0.00,0.13] | | |  | 0.18 [0.07,0.28] |  | 0.27 [0.18,0.35] |  | 0.37 [0.22,0.52] |  |
| Chair Away | 0.06 [0.01,0.12] | | |  | 0.24 [0.09,0.38] |  | 0.28 [0.11,0.45] |  | 0.34 [0.22,0.46] |  |
| Empty Chair | 0.20 [0.01,0.40] | | |  | 0.26 [0.10,0.42] |  | 0.20 [0.10,0.30] |  | 0.28 [0.16,0.40] |  |

**Part 3:** Analysis of confederate cell phone use in Experiment 2.

We examined if cell phone use by the confederate interacted with any of our effects in Experiment 2 using a Condition (load, no load) by Proximity (near, far), by Location (head, body), by Cell phone use (Cell; present, absent) mixed ANOVA on the proportion of fixations and the proportion of time spent fixating on the confederate. This analysis was exploratory and therefore these results are limited in what they can tell us about cell phone use and looking behaviour in our study.

*Proportion of Fixations.* There was no main effect of Cell Phone use, *F*(1,90)=0.11, *p*=.74, η^2^_g_=.00, BF_inc_=0.15. Cell phone use did not interact with Condition, *F*(1,90)=0.05, *p*=.82, η^2^_g_=.00, BF_inc_=0.20, Proximity, *F*(1,90)=0.05, *p*=.82, η^2^_g_=.00, BF_inc_=0.17, or Location, *F*(1,90)=0.94, *p*=.34, η^2^_g_=.01, BF_inc_=0.26. There was no Condition by Proximity by Cell Phone use interaction, *F*(1,90)=0.36, *p*=.55, η^2^_g_=.00, BF_inc_=0.24, as well as no Condition by Location by Cell Phone use interaction, *F*(1,90)=0.75, *p*=.39, η^2^_g_=.00, BF_inc_=0.40, and no Proximity by Location by Cell Phone interaction, *F*(1,90)=0.05, *p*=.83, η^2^_g_=.00, BF_inc_=0.26. The 4-way Condition by Proximity by Location by Cell Phone use interaction was also not significant, *F*(1,90)=2.35, *p*=.13, η^2^_g_=.01, BF_inc_=0.41, see Table S2.

*Proportion of Fixations.* There was no main effect of Cell Phone use, *F*(1,90)=0.07, *p*=.80, η^2^_g_=.00, BF_inc_=0.14. Cell Phone use did not interact with Condition, *F*(1,90)=0.31, *p*=.57, η^2^_g_=.00, BF_inc_=0.22, Proximity, *F*(1,90)=0.00, *p*=.99, η^2^_g_=.00, BF_inc_=0.16, or Location, *F*(1,90)=0.62, *p*=.43, η^2^_g_=.00, BF_inc_=0.25. There was no Condition by Proximity by Cell Phone use interaction, *F*(1,90)=0.39, *p*=.53, η^2^_g_=.00, BF_inc_=0.26 as well as no Condition by Location by Cell Phone use interaction, *F*(1,90)=0.68, *p*=.41, η^2^_g_=.00, BF_inc_=0.38, and no Proximity by Location by Cell Phone interaction, *F*(1,90)=0.63, *p*=.43, η^2^_g_=.00, BF_inc_=0.29. The 4-way Condition by Proximity by Location by Cell Phone use interaction was also not significant, *F*(1,90)=2.75, *p*=.10, η^2^_g_=.01, BF_inc_=0.69, see Table S2.

Table S2. *Means and 95% confidence intervals for the use of cellphones by confederates in Experiment 2 for both proportion of fixations and proportion of time, across Condition, Proximity and Location.*

|  | **Load** | | | | |  | **No Load** | | | | |
| --- | --- | --- | --- | --- | --- | --- | --- | --- | --- | --- | --- |
|  | **Near** | |  | **Far** | |  | **Near** | |  | **Far** |  |
|  | Head | Body |  | Head | Body |  | Head | Body |  | Head | Body |
| ***Proportion of Fixations*** | | | | | | | | | | | |
| Cell Phone use | 0.07 [0.03,0.11] | 0.10 [0.04,0.15] |  | 0.07 [0.02,0.13] | 0.18 [0.10,0.25] |  | 0.21 [0.13,0.29] | 0.13 [0.06,0.19] |  | 0.12 [0.07,0.18] | 0.14 [0.07,0.22] |
| No Phone use | 0.05 [-0.01,0.10] | 0.12 [0.01,0.24] |  | 0.08 [0.03,0.14] | 0.15 [0.05,0.25] |  | 0.18 [0.09,0.28] | 0.11 [0.04,0.19] |  | 0.06 [0.02,0.09] | 0.21 [0.11,0.32] |
| ***Proportion of Time*** | | | | | | | | | | | |
| Cell Phone use | 0.04 [0.01,0.07] | 0.08 [0.03,0.14] |  | 0.10 [0.02,0.17] | 0.20 [0.11,0.29] |  | 0.23 [0.13,0.32] | 0.14 [0.06,0.22] |  | 0.15 [0.08,0.22] | 0.15 [0.07,0.23] |
| No Phone use | 0.04 [-0.00,0.09] | 0.11 [-0.01,0.24] |  | 0.11 [0.05,0.17] | 0.18 [0.06,0.30] |  | 0.20 [0.09,0.31] | 0.11 [0.03,0.19] |  | 0.06 [0.02,0.10] | 0.23 [0.11,0.35] |
